# A Novel Animal Model of *Borrelia recurrentis* Louse-Borne Relapsing Fever Borreliosis Using Immunodeficient Mice

**DOI:** 10.1371/journal.pntd.0000522

**Published:** 2009-09-29

**Authors:** Christer Larsson, Jenny Lundqvist, Nico van Rooijen, Sven Bergström

**Affiliations:** 1 Umeå University, Department of Molecular Biology and Laboratory for Molecular Infection Medicine Sweden (MIMS), Umeå, Sweden; 2 Vrije University, Department of Molecular Cell Biology, Amsterdam, The Netherlands; Weill Medical College of Cornell University, United States of America

## Abstract

Louse-borne relapsing fever (LBRF) borreliosis is caused by *Borrelia recurrentis*, and it is a deadly although treatable disease that is endemic in the Horn of Africa but has epidemic potential. Research on LBRF has been severely hampered because successful infection with *B. recurrentis* has been achieved only in primates (i.e., not in other laboratory or domestic animals). Here, we present the first non-primate animal model of LBRF, using SCID (-B, -T cells) and SCID BEIGE (-B, -T, -NK cells) immunocompromised mice. These animals were infected with *B. recurrentis* A11 or A17, or with *B. duttonii* 1120K3 as controls. *B. recurrentis* caused a relatively mild but persistent infection in SCID and SCID BEIGE mice, but did not proliferate in NUDE (-T) and BALB/c (wild-type) mice. *B. duttonii* was infectious but not lethal in all animals. These findings demonstrate that the immune response can limit relapsing fever even in the absence of humoral defense mechanisms. To study the significance of phagocytic cells in this context, we induced systemic depletion of such cells in the experimental mice by injecting them with clodronate liposomes, which resulted in uncontrolled *B. duttonii* growth and a one-hundred-fold increase in *B. recurrentis* titers in blood. This observation highlights the role of macrophages and other phagocytes in controlling relapsing fever infection. *B. recurrentis* evolved from *B. duttonii* to become a primate-specific pathogen that has lost the ability to infect immunocompetent rodents, probably through genetic degeneration. Here, we describe a novel animal model of *B. recurrentis* based on B- and T-cell-deficient mice, which we believe will be very valuable in future research on LBRF. Our study also reveals the importance of B-cells and phagocytes in controlling relapsing fever infection.

## Author Summary

Research on *Borrelia recurrentis*, the agent of louse-borne relapsing fever (LBRF), has been hampered by the lack of a feasible non-primate animal model. By using immunocompromised SCID mice deficient in B- and T-cells, we were able to establish a stable, persistent *B. recurrentis* infection with low spirochetemia. Furthermore, systemic depletion of phagocytes by use of clodronate liposomes increased the numbers of bacteria in blood, which demonstrates the importance of both the humoral response and phagocytosis in controlling relapsing fever infection. Lice are favored by the conditions related to the unfortunate turmoil and refugee camps prevailing in the Horn of Africa, and hence LBRF is more important now than it has been for several decades. The newly published genome sequence of *B. recurrentis* and techniques to genetically manipulate RF borreliae will be instrumental in understanding its complex biology. We therefore believe that our novel animal model will be a great asset that can facilitate future studies of the infection biology of *B. recurrentis*.

## Introduction

Bacteria of the genus *Borrelia* are spirochetes that cause either Lyme disease or relapsing fever (RF). *Borrelia* species are transferred from animals to humans by tick bites, with the single exception of *B. recurrentis*, which is transmitted between humans by the body louse *Pediculus humanus humanus*. The louse-borne disease is not transmitted by the bite *per se*, but rather through contamination of abraded skin by feces or coelomic fluid released from lice that are crushed by scratching. *P. humanus humanus* is strictly a human-specific parasite that lives on the body and in the clothing of its host, and *B. recurrentis* has been found only in lice and humans [1],[2].

In most cases, louse-borne relapsing fever (LBRF) presents with a sudden onset of fever (typically 38.7–41°C) and chills. The first fever period lasts on average 5–7 days and is accompanied by malaise, nausea, general aches, and enlargement of the spleen and liver. Compared to tick-borne RF, LBRF usually involves fewer relapses, but it results in far greater mortality, which can be as high as 40% if left untreated but as low as 1%–5% when antibiotic therapy is given. The Jarish-Herxheimer reaction and delayed onset of antimicrobial therapy are associated with an elevated mortality risk [1],[2],[3].

LBRF previously occurred worldwide in massive epidemics, the latest of which were seen during the two world wars. Today, the only endemic area is in the highlands of Ethiopia, and sporadic outbreaks have been observed in Sudan, where the disease is associated with natural disasters, famine, and refugee camps [1],[2],[4],[5],[6]. Although *B. recurrentis* is currently found only in the Horn of Africa, it can become established wherever there are human body lice and thus it has a high potential to cause global epidemics [7], especially today due to the massive political turmoil in the Horn of Africa. A population of lice can increase by 11% a day, which gives a clue as to just how rapidly an outbreak can spread in places like refugee camps [1],[8]. Therefore, LBRF may be even more important now than it has been for decades.

Despite increasing epidemic potential and important scientific progress such as the recent genome sequencing of *B. recurrentis* [9], research is hampered by the lack of feasible animal models. In the early 20th century, scientists attempted to infect commonly used laboratory animals as well as several species of domestic and wild animals, but only experiments using primates seemed to have been successful [10]. Moreover, *B. recurrentis* was not isolated *in vitro* until 1994, when Cutler and coworkers [11] managed to grow a few strains in BSK broth. That event paved the way for microbiological and biochemical investigations, but the lack of an animal model has continued to hinder performance of all types of studies focused on host-pathogen interactions, as well as other experiments that require an *in vivo* model system.

We used immunodeficient mouse strains to develop an animal model of *B. recurrentis* infection that is more practical in all aspects compared to a primate-based model. SCID mice carry the *Prkdc^scid^* mutation, which results in severe combined immunodeficiency due to a defect in V(D)J recombination; this condition impairs the animals' ability to generate B- and T-cell antigen receptors, thus leading to very low numbers of functional lymphocytes [12]. NUDE mice lack a thymus, and they have impaired T-cell function due to the *Foxn1^nu^* mutation [13], whereas the cells of their innate immune system (monocytes, macrophages, natural killer cells and neutrophils) and their complement system remain functional. Mice with the BEIGE mutation (*Lyst^bg^*) have defective natural killer (NK) cells [14]. Hence, SCID BEIGE mice lack B-, T-, and NK-cells.

Clodronate liposomes have been used extensively in infection models to study the pathobiological effect of systemic depletion of phagocytic cells (e.g., macrophages) and the immunological importance of such cells in combating infection [15],[16],[17],[18]. These liposomes are artificially prepared lipid vesicles that encapsulate clodronate, and they can be injected intravenously to attack phagocytic cells that are present in or in contact with the blood (e.g., macrophages in the spleen and liver). The clodronate is ingested by and accumulated within phagocytic cells, and after an intracellular threshold concentration of the drug is exceeded, the cells are irreversibly damaged and die by apoptosis, as described elsewhere [19]. Still, it is important to remember that clodronate does not eliminate all phagocytic cells, and that new phagocytes will continuously appear as soon as the liposomes are consumed; in other words, the phagocytic activity cannot be totally inhibited. Free clodronate (i.e. released from dead macrophages) has a very short half-life and is quickly removed from the circulation by the renal system, furthermore it can not easily pass through cell membranes and thus can not affect non-phagocytic cells [20].

By using animals deficient in various immune cells and inducing systemic depletion of phagocytes, much can be learned about the immune defense against RF borreliosis and host-pathogen interactions. In the current investigation, we found that SCID mice could support growth of *B. recurrentis*, which led to a low-grade, persistent disease. Moreover, employing clodronate liposomes to deplete phagocytic cells resulted in a one-hundred-fold rise in the spirochete titers. Thus, in this paper we present the first non-primate animal model for studies of LBRF. We also characterize *B. recurrentis* infection and compare it with the closely related species *B. duttonii*, which is virulent in wild-type mice and has been studied extensively in mouse models [21],[22].

## Methods

### Bacterial strains and growth

The two *B. recurrentis* strains A11 and A17 were isolated in Ethiopia and were kindly provided by Sally Cutler, University of East London. Isolation and characterization of these strains has been described by other investigators [11]. *B. duttonii* 1120K3 was kindly provided by Guy Baranton, Institute Pasteur. All bacteria were cultured at 37°C in BSK-II medium supplemented with 10% (v/v) rabbit serum and 1.4% (w/v) gelatin, as described elsewhere [23]. The bacteria used in experiments had less than 10 passages through animals or *in vitro* in our lab.

### Animal infection

To create and validate a *B. recurrentis* non-primate animal model, we injected 1×10^6^ *B. duttonii*, *B. recurrentis* A11, or *B. recurrentis* A17 subcutaneously (s.c.) into four 6-week-old male mice of each of the following strains (all from Taconic, Denmark): BALB/c (BALB/cAnNTac), BALB/c NUDE (C.Cg/AnNTac-*Foxn1^nu^* NE9), SCID (C.B-*Igh-1^b^*/IcrTac-*Prkdc^scid^*), and SCID BEIGE (C.B-*Igh*-1b/GbmsTac-*Prkdc^scid^-Lyst^bg^* N7). The animals were kept in a filter cabinet and given food and water *ad libitum*, with all maintenance performed according to Swedish animal welfare guidelines. Tail blood was collected daily, and bacteria in the samples were counted by phase contrast microscopy during the first 20 days of infection. SCID and SCID BEIGE mice infected with *B. recurrentis* were kept until day 150 post infection (p.i.), and spirochetemia was quantified weekly by microscopy. All animal experiments were approved in advance by the Laboratory Animal Ethics Committee of Umeå University.

### Depletion of phagocytic cells

Starting one day before infection and subsequently every fifth day, an intravenous (i.v.) injection of 100 µl clodronate liposomes in phosphate-buffered saline (PBS) was administered in the tail of SCID BEIGE and BALB/c mice to deplete phagocytic cells present in the blood and organs (e.g., spleen and liver); this was done as described elsewhere [19]. Since the phagocytes might also have ingested liposomes that contained PBS instead of clodronate, which would have reduced the phagocytic efficiency, we gave mice i.v. injections of 100 µl PBS as negative controls. The animals were infected with either *B. recurrentis* A17 or *B. duttonii* 1120K3 five days after the first clodronate injection.

### Statistics

Spirochetemia (non-Gaussian) was analyzed by the Mann-Whitney U-test, and the results are presented as medians with 25th and 75th percentile bars to illustrate variance. Difference in spleen weight was assessed by Student's t-test.

## Results

**Figure 1:**
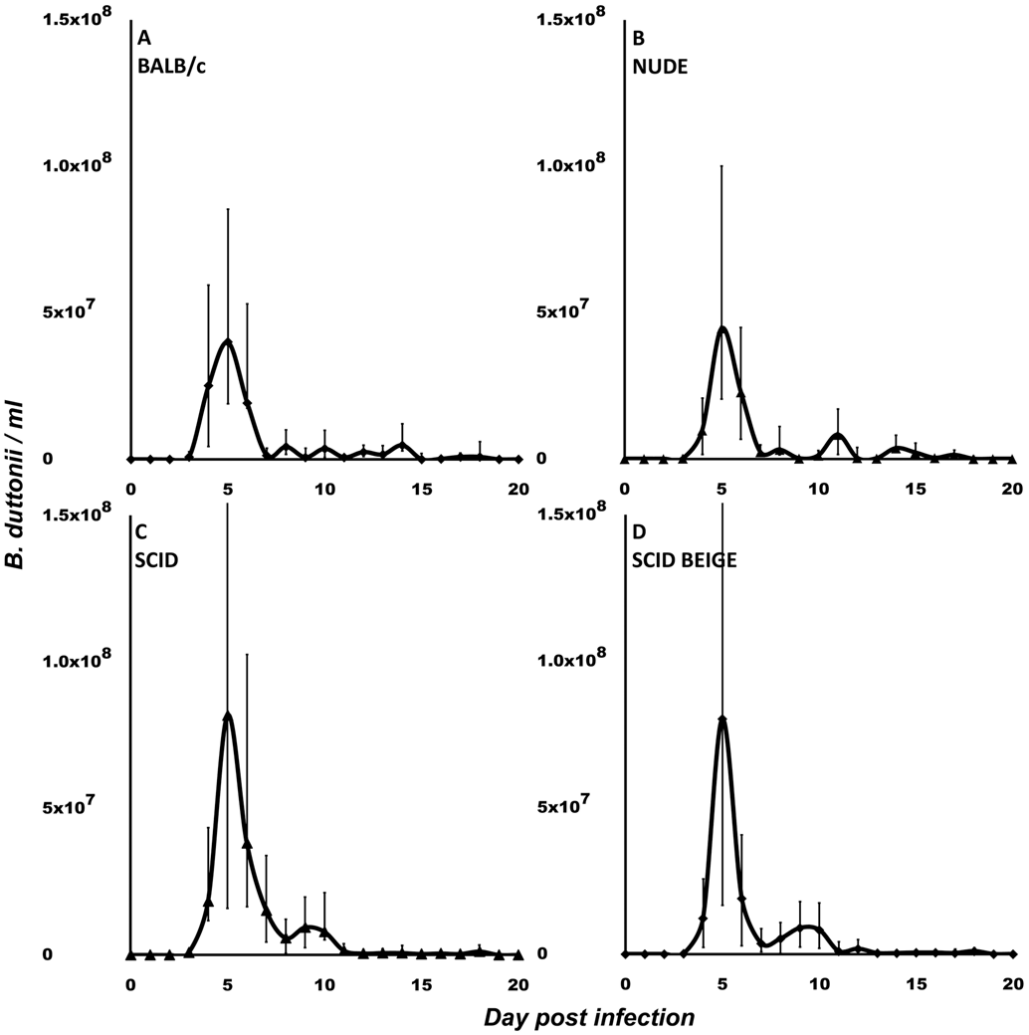
*B. duttonii* spirochetemia in mice. BALB/c (A), NUDE (B), SCID (C), and SCID BEIGE (D) mice. *B. duttonii* established infection in all four mouse strains. Spirochetemia was significantly (p = 0.02) higher in B-cell-deficient mice (C, D). No differences were observed between wild-type and T-cell-deficient mice (A vs. B) or between SCID mice and NK-cell-deficient SCID BEIGE mice (C vs. D). Data are presented as medians with 25^th^ and 75^th^ percentiles. Notice that the scales of the y-axes in this figure differ from those in Figures 2 and 4.

**Figure 2:**
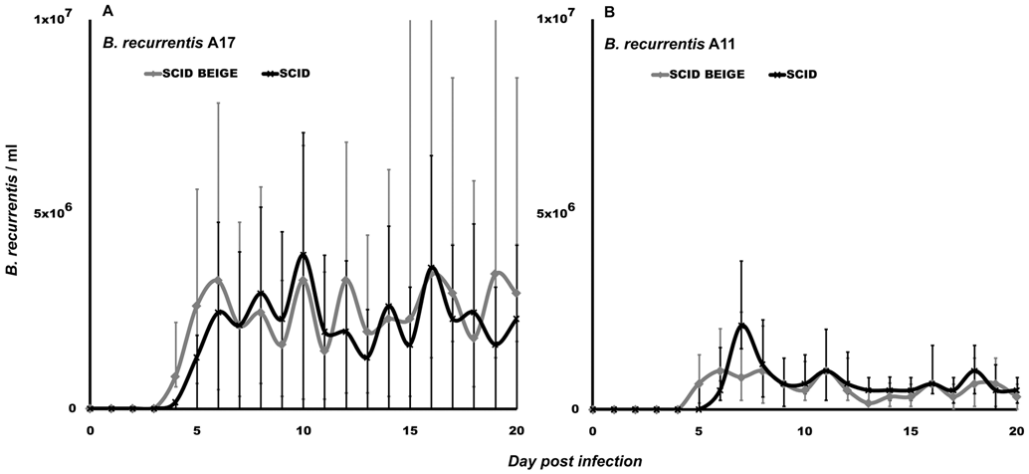
*B. recurrentis* spirochetemia in SCID BEIGE and SCID mice. *B. recurrentis* established a relatively low-grade (compared to *B. duttonii*, see Figure 1C, D), stable infection in SCID BEIGE and SCID mice, but not in BALB/c or NUDE animals. Strain A17 (panel A) caused a higher (p<0.01) level of spirochetemia than did strain A11 (panel B). No difference in *B. recurrentis* spirochetemia was observed between SCID BEIGE and SCID mice. Data are presented as medians with 25^th^ and 75^th^ percentiles.

**Figure 3:**
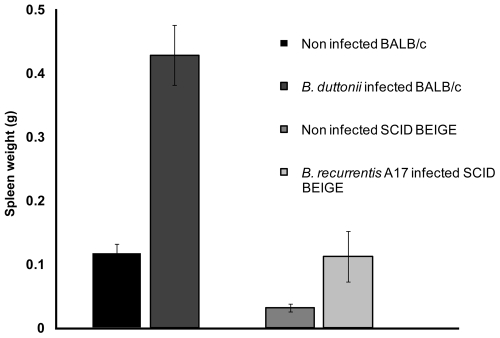
Splenomegaly in infected mice. Although expansion of B- and T-cells is the major cause of splenomegaly in wild-type animals, SCID BEIGE mice infected with *B. recurrentis* A17 displayed significant (p<0.01) splenomegaly,which indicates a T-, B-, and NK-cell-independent immune response.

We conducted tests to determine whether any of the commercially available immunodeficient mouse strains can support growth of *B. recurrentis*. Initially, the two *B. recurrentis* strains A11 and A17, and *B. duttonii* strain 1120K3 were inoculated into immunocompetent wild-type BALB/c mice, NUDE mice lacking T-cells, SCID mice lacking B- and T-cells, and SCID BEIGE mice lacking B-, T-, and NK-cells. As expected, only *B. duttonii* established detectable infection in the BALB/c mice (Figure 1A), which concurred with the results of similar previous experiments [22],[24]. The total *B. duttonii* spirochetemia did not differ significantly between NUDE mice and BALB/c mice (Figure 1A–B), and neither the A11 nor the A17 *B. recurrentis* strain caused detectable spirochetemia in BALB/c or NUDE mice, indicating that T-cells are of minor importance for RF immune defense. However, both the *B. recurrentis* strains did establish infection in SCID and SCID BEIGE mice, although the spirochetemia was about 200-fold lower than that caused by *B. duttonii* infection (Figures 1 C–D and 2). Due to the lack of antibodies, *B. recurrentis* spirochetemia did not display a relapsing pattern. Instead, spirochete titers remained fairly constant over time, and there were only minor fluctuations between the mice, which were probably caused by individual, biological variations in the host-pathogen interactions (Figure 2). Both the A11 and A17 bacteria were persistent and remained at fairly low levels in the blood (about 2×10^5^/ml) at least until day 150 post infection. The A11 strain caused a significantly (p<0.01) milder infection, with spirochetemia about half the magnitude of that caused by the A17 strain in both SCID and SCID BEIGE mice (Figure 2). The animals behaved normally and showed no outer sign of disease, and their weight pattern corresponded to what was seen in uninfected animals (data not shown). To ascertain whether the passage through SCID BEIGE mice rendered the bacteria capable of causing spirochetemia in wild-type mice, we inoculated four BALB/c mice with spirochetes obtained from two A11-infected and two A17-infected animals. None of those four mice developed detectable spirochetemia. The *B. recurrentis* A17 infection induced significant (p<0.01) splenomegaly in SCID BEIGE mice. However, the spleens were much smaller in B-cell- and T-cell-deficient mice than in wild-type mice (Figure 3), which implies activation and multiplication of splenic immune cells. Surprisingly, *B. duttonii* did not cause a lethal infection in the B-cell-deficient animals, despite the indispensable role of B-cells suggested by the antibody-mediated clearance of antigenic variants in immunocompetent models (Figure 1C, D). Although both *B. recurrentis* and *B. duttonii* spirochetemia were higher in B-cell-deficient mice (p = 0.02), which verifies significance of the B-cells, the disease was kept under control by B-cell-independent mechanisms (Figures 1C, D and 2).

**Figure 4:**
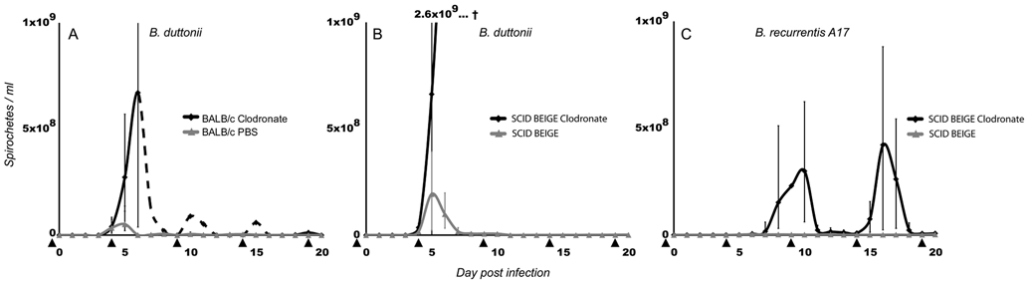
Effect of clodronate-induced macrophage depletion on spirochetemia. Mice were injected with clodronate liposomes in PBS to deplete macrophages (and other phagocytic cells) in the blood and in organs in contact with the blood (e.g., the spleen and liver). PBS was used as a negative control. The injections (indicated by black triangles) were given every fifth day, starting one day before infection. A. BALB/c mice infected with *B. duttonii* developed very high spirochetemia, and all except one individual (dotted line) died at day 6 p.i. B. *B. duttonii*-infected SCID BEIGE mice treated with clodronate developed uncontrolled spirochetemia, and they all died before day 8 p.i. Control SCID BEIGE and BALB/c mice injected with PBS had much lower spirochetemia compared to their macrophage-depleted counterparts. C. SCID BEIGE mice treated with clodronate and infected with *B. recurrentis* A17 developed high but not lethal spirochetemia. The maximum spirochetemia in any of the control SCID BEIGE and BALB/c mice injected with PBS was 7.3×10^6^/ml. Even between the two peaks shown for the *B. recurrentis*-infected, clodronate-treated mice, the median spirochetemia was never below 8×10^6^/ml. *B. recurrentis* was unable to establish detectable spirochetemia in macrophage-depleted BALB/c mice.

Notably, we also found that clodronate-treated (i.e., phagocyte-depleted) mice infected with *B. duttonii* were unable to restrict the bacterial infection. These animals developed very high spirochetemia, which killed all the SCID BEIGE mice before day 8 p.i. (Figure 4B). The pattern was similar in BALB/c mice, although one single individ survived and managed to control the infection (Figure 4A). *B. recurrentis* A17 also caused substantial spirochetemia in clodronate-treated SCID BEIGE mice (Figure 4C), although the level that was reached was about 10 times higher than the spirochetemia induced by *B. duttonii* in untreated wild-type mice (Figure 4A and 4C). However, *B. recurrentis* was unable to establish infection in phagocyte-depleted BALB/c mice, which underlines the significance of B-cells and possibly also T-cells.

## Discussion

Several attempts have been made to establish *B. recurrentis* infection in various animals, but, until now, only primate models have been successful [10], which has severely hampered research on LBRF. In the present study, we used mice deficient in T- and B-cells and induced phagocyte depletion by administering clodronate liposomes to create the first non-primate animal model of LBRF infection. The results of our experiments show that *B. recurrentis* could infect both SCID and SCID BEIGE mice. The two *B. recurrentis* strains A11 and A17 caused moderate, persistent infection in B- and T-cell-deficient SCID mice and B-, T- and NK-cell-deficient SCID BEIGE mice (Figure 2), but did not induce detectable spirochetemia in either wild-type BALB/c animals or NUDE mice lacking mature T-cells. The A11 and A17 strains we used were isolated from different LBRF patients in Ethiopia [11] and had an estimated history of ∼20 BSK passages. Since RF spirochetes do not lose plasmids *in vitro* (which is the case in Lyme *Borrelia* spirochetes), and they maintain infectivity even after several passages *in vitro* [9],[25], the two strains we chose to use were probably well representative of *B. recurrentis*.

Despite the ability of RF spirochetes to evade the host humoral response through antigenic variation, antibodies are definitely an important part of immune defense against this disease [26],[27]. This is also reflected by the present findings showing higher *B. duttonii* spirochetemia and establishment of *B. recurrentis* infection in the B-cell-deficient mice. Moreover, the interaction between the humoral response and *B. recurrentis* seems to be somewhat different than noted for other RF-inducing bacteria, since *B. recurrentis* generally causes 0–4 relapses, whereas other African species cause 3–9, as reviewed by other researchers [1],[2]. T-cells are apparently less important, since we observed that *B. duttonii* spirochetemia was equally high in wild-type BALB/c animals and the athymic, T-cell-deficient NUDE mice (Figure 1A, B). Similar results have been reported in experiments on both the RF agent *B. turicatae* and Lyme borreliosis [28],[29]. Furthermore, an investigation of RAG2^−/−^ and RAG2/IL-10^−/−^ mice infected with *B. turicatae* has suggested that NK-cells play an important role [30]. In contrast, we found that SCID mice with the BEIGE mutation, which causes NK-cell deficiency, showed the same levels of *B. recurrentis* or *B. duttonii* spirochetemia as seen in SCID mice (Figures 1C, D and 2). On the other hand, phagocyte depletion by use of clodronate liposomes had a dramatic effect on both spirochetemia and disease. *B. duttonii* infection was uncontrollable in all animals but one, and even *B. recurrentis* reached spirochetemia of over 5×10^8^/ml. The apparent “relapses” in clodronate-treated animals were not due to antigenic variation, but instead to partial host recovery from the phagocytic depletion that was repeated every fifth day (Figure 4A, C). Even between the two peaks in *B. recurrentis*-infected mice, median spirochetemia never receded below 8×10^6^/ml, which is a fairly high level (Figure 4C). These results clearly illustrate that it is important for phagocytic cells to be able to filter and to some extent also control spirochetes in the blood, even in the absence of B- and T-cells. Furthermore, experiments *in vitro* have indicated that *B. recurrentis* avoids complement opsonization and phagocytosis by binding complement regulators such as factor H and by degrading C3b from its surface in order to utilize “hijacked” host plasmin [31],[32]. However, this remains to be convincingly demonstrated *in vivo*. Despite all of these isolated findings, it is essential to bear in mind that the immune system is a tightly connected web of cells and signal molecules, and that removal of one cell type might have other downstream effects on overall immunity.

The recent genome analysis performed by Lescot et al. [9] revealed that *B. recurrentis* evolved from *B. duttonii* through extensive genetic decay, probably involving loss of the genes *mutS* and *recA*, which are important for DNA repair. Both of these species are considered to be more or less specific to humans, and it has been debated whether this is due to a restricted host infectivity range, or simply to the fact that both the *B. duttonii* tick vector *Ornithodorus moubata moubata* and the *B. recurrentis* louse vector *P. humanus humanus* strongly prefer humans as a source of food. *B. duttonii* establishes RF in wild-type laboratory mice and rats that is similar to the human disease, and this spirochete has recently been found in chickens and pigs raised in proximity to tick-infested human dwellings in Tanzania [33], indicating a wider potential host range than was previously believed. In short, *B. duttonii* can infect different species of animals, but it is ecologically very restricted by the host range of its vector. It might be assumed that *B. recurrentis* should also be able to infect non-primate animal species, perhaps even more severely than does *B. duttonii*, since in humans it is basically a more pathogenic strain of *B. duttonii*. Interestingly, this greater virulence seems to originate from the loss of a gene or genes rather than the gain of novel genes [9], as has also been described in other louse-borne infections, such as *Rickettsia prowazekii* [34]. *B. recurrentis* has lost its ability to survive in hosts other than humans, and the way this spirochete is transmitted (i.e., not by the louse bite but by fecal or hemolymph contamination of abraded skin) is a somewhat unsophisticated strategy that suggests a short evolutionary adaptation. It is tempting to speculate that the lost capacity of *B. recurrentis* to infect non-primate animals may also be a result of genomic decay. *B. duttonii* probably possesses mechanisms for evading the immune defense of host animals, which have disappeared in *B. recurrentis* during its rapid evolution simply because they are not needed since humans are the only host. Inasmuch as SCID mice are defective in B- and T-cell production, they readily accept foreign cells and tissues without rejection. Future experiments by our group should be aimed at creating humanized SCID-hu mice that generate human immune cells or carry stem-cell-producing human xenotransplants to pinpoint the factors that restrict LBRF to humans. Such strategies have been successfully applied in other human-specific infectious diseases, as described by other investigators [35]. For instance, SCID-hu mice with human B-cells may constitute a better model of human *B. recurrentis* infection that can facilitate studies of aspects such as antigenic variation that seems to be responsible for a difference causing fewer relapses compared to tick-borne RF [2].

*B. recurrentis* was grown *in vitro* for the first time in 1994 [11], which opened the door for microbiological and immunological studies conducted *in vitro* [32],[36]. In addition, the complete genomes of *B. recurrentis* and *B. duttonii* were recently sequenced and published for the first time [9], and the first site-specific genetic manipulation of an RF agent was performed last year when the variable tick protein was knocked out and reconstituted in *B. hermsii* [37]. All of these achievements will definitely encourage further development of genetic tools and facilitate molecular biological investigations of RF. Here, we have described the first non-primate animal model of LBRF, which we believe is the fourth cornerstone needed to bring *B. recurrentis* research into the 21st century.
